# Bring the past to the future: adapting stereoscope images for use in the Oculus Go

**DOI:** 10.5195/jmla.2020.1039

**Published:** 2020-10-01

**Authors:** Dorothy C. Ogdon, Stefanie Crumpton

**Affiliations:** 1 dogdon@uab.edu, Emerging Technologies Librarian, Libraries, University of Alabama at Birmingham, 250-B Lister Hill Library, 1720 Second Avenue South, Birmingham, AL 35294; 2 scrumpton@uab.edu, Curator, Alabama Museum of the Health Sciences, Libraries, University of Alabama at Birmingham, 1720 Second Avenue South, Birmingham, AL 35294

## Abstract

The purpose of this project was to explore methods for adapting images originally created for the analog stereoscope to use in contemporary virtual reality headsets. The Alabama Museum of the Health Sciences holds in its collections a set of medical images for the stereoscope published by Dr. S.I. Rainforth in 1910. We scanned 3 stereoscope cards from the collection at a resolution of 1200 dots per inch, then adapted the images for use in virtual reality using Adobe Photoshop and Unity. We successfully created a working application for the Oculus Go that displays the images stereoscopically in the headset. The current application allows only static display of the images. Our next steps in developing this project will be to add additional images from the collection to the virtual reality application, optimize parameters related to image display, and develop scripting that would allow users to dynamically select images from the collection. More information on this project is available on the Alabama Museum of the Health Sciences Virtual Exhibits website, and a short video demonstration is available on Vimeo.

The University of Alabama at Birmingham (UAB) Libraries have been working to build capacity in emerging areas such as three-dimensional (3D) printing and immersive reality. The Alabama Museum of the Health Sciences (AMHS) at the UAB Libraries preserves material related to diagnosis of diseases, therapeutic and treatment successes, struggles at the UAB Medical Center, the practitioners on campus, and those they have trained. This project is the first attempt in the library to adapt objects from a museum collection for use in virtual reality.

Perhaps prophetically, in the 2018 academic year, 4 UAB students used pieces from the AMHS and neighboring Reynolds-Finley Historical Library to satisfy requirements for their senior projects and created an exhibition titled *Scourge: Diseases that Shaped History*. The exhibition included cards from *The Stereoscopic Skin Clinic*, first published by Dr. [Seldon Irwin] S.I. Rainforth in 1910. *The Stereoscopic Skin Clinic* is a set of more than 100 photographs featuring several skin diseases in a stereoscope format. Cards from 2 bundles of *The Stereoscopic Skin Clinic* were donated to the museum in 1997. In addition to the cards, the AMHS also holds 6 different models of stereoscopes in its collections (Figure 1), with 2 designed to hold the cards effectively and only 1 with complete hardware to do so.

**Figure 1:**
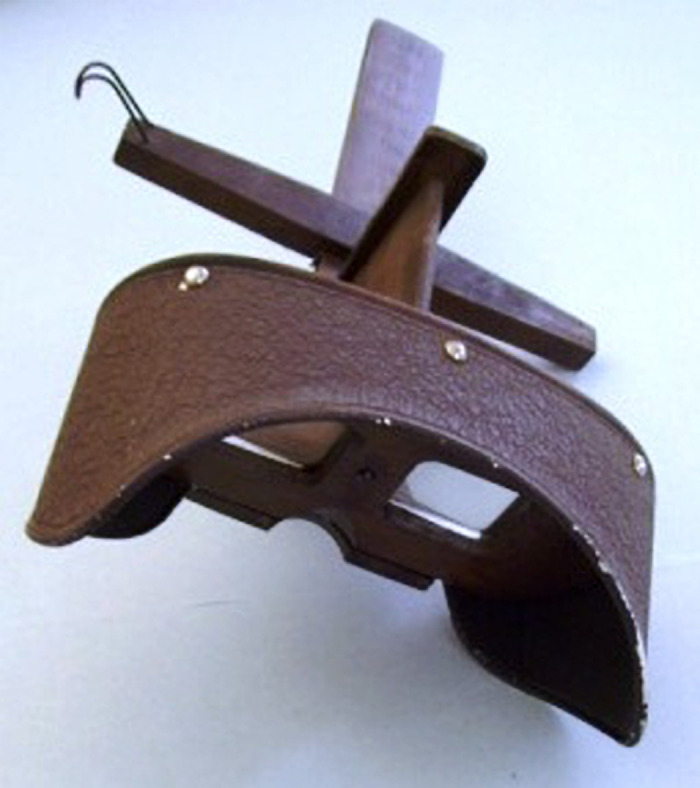
A stereoscope from the collections of the Alabama Museum of the Health Sciences

The authors' visit to the *Scourge* exhibit in early 2019 generated the question of whether the stereoscope cards could be adapted for use in virtual reality headsets. While researching the question, we found a tutorial published by Loren Abdulezer in 2015 [1] that helped form the technical foundation of this project. The tutorial describes methods for rendering images stereoscopically in Unity [2], a popular software package that provides development tools for virtual reality applications. The AMHS curator selected three cards with stereoscopic photographs of hands affected by scabies, variola, and eczema (Figure 2) from *The Stereoscopic Skin Clinic* to be the image test set and digitized the front and back of each card using an Epson flatbed scanner. We used image-cropping and editing tools in Photoshop to prepare separate right and left images from each scanned card for use in Unity. The separate right and left images that we created using Photoshop are a uniform size, 2726 pixels wide by 3856 pixels high and have an 800 pixel-per-inch resolution.

**Figure 2:**
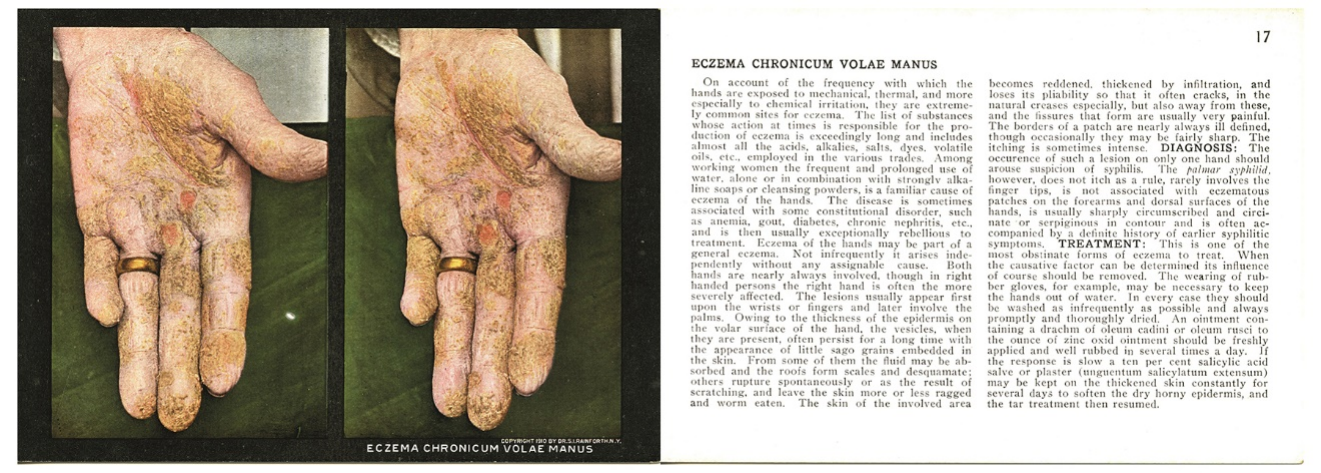
Front and back of the stereoscope card labeled “Eczema Chronicum Volae Manus” from *The Stereoscopic Skin Clinic* published by Dr. [Seldon Irwin] Rainforth in 1910

Once we completed the image preparation, we imported an initial set of right and left images into Unity to test display settings and made adjustments as needed. We used Unity to produce an application file that was suitable for the Oculus Go virtual reality headset. The Oculus Go had a low price point and did not require the use of external sensors, making it easy to use in environments with space limitations such as the AMHS public gallery. We installed the application on the headset using a process known as side-loading, which required use of the headset's developer mode [3]. We can now display the scanned images stereoscopically in the Oculus Go headset by launching the application that we created.

We are encouraged by the success of our initial exploration of adapting images from *The Stereoscopic Skin Clinic* for use in a virtual reality headset. This use of a twenty-first century technology to simulate a nineteenth century concept caused no harm to the pieces and vibrantly enhanced the original purpose of the pieces in the translation for use in a virtual reality headset. Users who have informally tested the application have been enthusiastic but have found the variations in lighting and the readability of the descriptive text for each card challenging (Figure 3). We plan to explore methods for providing this information in alternative formats such as audio recordings that can be played in the application. In future iterations, we also plan to address the usability challenge that the initial iteration of the application lacks interactive controls and relies on users changing the orientation of their heads or bodies to view each image.

**Figure 3:**
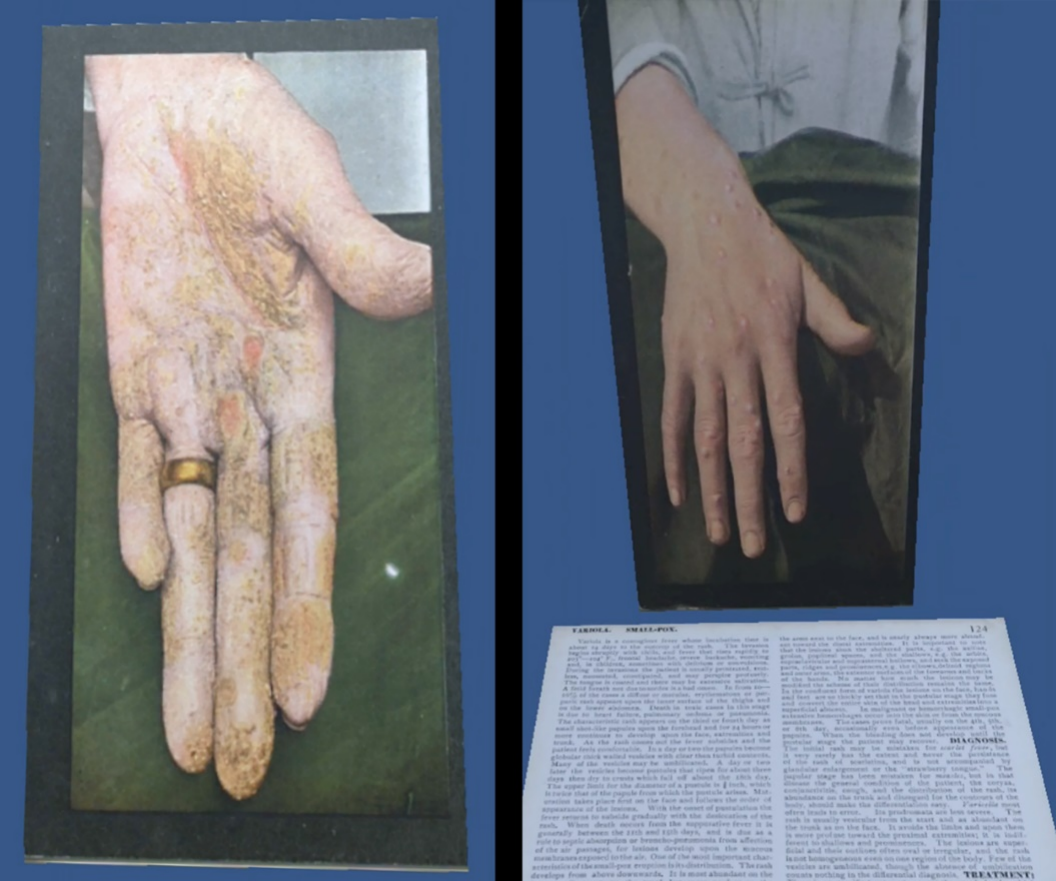
A still image of the stereoscope cards labeled “Eczema Chronicum Volae Manus and Variola,” as rendered in the Oculus Go virtual reality headset

The images as captured do not demonstrate stereoscopic rendering because the Oculus Go tools for capturing video only capture information displayed in the left screen in the headset.

As of June 2020, the AMHS remained closed to visitors due to the COVID-19 pandemic. The museum has developed a virtual exhibit with information on this project, in addition to its other virtual offerings. While access to the physical museum space is limited, we plan to continue refining the functionality of this application and anticipate adding scans for stereoscopic rendering once the physical collections can be accessed again. Whenever the AMHS reopens, there is space for the museum gallery to expand to an adjacent similar-sized room, which could allow more permanent and safer interactivity with items like the virtual reality renderings of *The Stereoscopic Skin Clinic.*
